# The early postnatal period: Exploring women's views, expectations and experiences of care using focus groups in Victoria, Australia

**DOI:** 10.1186/1471-2393-8-27

**Published:** 2008-07-22

**Authors:** Della A Forster, Helen L McLachlan, Jo Rayner, Jane Yelland, Lisa Gold, Sharon Rayner

**Affiliations:** 1 Mother and Child Health Research, La Trobe University, 324-328 Lt Lonsdale St, Melbourne, Australia; 2 Royal Women's Hospital, Locked Bag 300, Grattan St and Flemington Rd, Parkville, 3052, Australia; 3 School of Nursing and Midwifery, La Trobe University, 3086, Australia; 4 Healthy Mothers, Healthy Families. Murdoch Children's Research Institute, PO Box 911, Parkville, 3052, Australia; 5 Health Economics Unit, School of Health and Social Development, Deakin University, 221 Burwood Highway, Burwood, 3125, Australia

## Abstract

**Background:**

There is growing evidence from Australia and overseas that the care provided in hospital in the early postnatal period is less than ideal for both women and care providers. Many health services face increasing pressure on hospital beds and have limited physical space available to care for mothers and their babies. We aimed to gain a more in-depth understanding of women's views, expectations and experiences of early postnatal care.

**Methods:**

We conducted focus groups in rural and metropolitan Victoria, Australia in 2006. Fifty-two people participated in eight focus groups and four interviews. Participants included eight pregnant women, of whom seven were pregnant with their first baby; 42 women who were in the postpartum period (some up to twelve months after the birth of their baby); and two partners. All participants were fluent in English. Focus group guides were developed specifically for the study and explored participants' experiences and/or expectations of early postnatal care in hospital and at home, with an emphasis on length of hospital stay, professional and social support, continuity of care, and rest. Discussions were audio-taped and transcribed verbatim. A thematic network was constructed to describe and connect categories with emerging basic, organizing, and global themes.

**Results:**

Global themes that emerged were: anxiety and/or fear; and the transition to motherhood and parenting. The needs of first time mothers were considered to be different to the needs of women who had already experienced motherhood. The women in this study were generally concerned about the safety of their new baby, and lacked confidence in themselves as new mothers regarding their ability to care for their baby. There was a consistent view that the physical presence and availability of professional support helped alleviate these concerns, and this was especially the case for women having a first baby.

**Conclusion:**

Women have anxieties and fears around early parenting and their changing role, and may consider that the physical availability of professional care providers will help during this time. Care providers should be cognisant of these potential issues. It is crucial that women's concerns and needs be considered when service delivery changes are planned. If anxiety around new parenting is a predominant view then care providers need to recognise this and ensure care is individualised to address each woman's/families particular concerns.

## Background

A decade of research in Victoria, Australia, has identified low levels of satisfaction with the hospital stay following birth. Of the three episodes of maternity care, women are least likely to be satisfied with postnatal care, with only 50% of women in a 2000 state-wide survey rating their postnatal care as 'very good'. In contrast, 67% and 72% rated antenatal care and intrapartum care respectively as 'very good' [1]. The factors most strongly associated with negative ratings of postnatal care were those reflecting women's experiences of specific aspects of care: the sensitivity of caregivers; the extent to which anxieties and concerns were taken seriously; how rushed caregivers seemed; the helpfulness of advice and support; and whether help and advice were offered at all [1]. Length of postnatal hospital stay was also associated with satisfaction with care; staying in hospital for one to two days was associated with less positive ratings of care, compared with the views of women who stayed five days or more [1].

A recently completed state-wide review of public hospital postnatal care from the providers' perspective (the PinC review) identified that postnatal care provision was both diverse and complex [2]. There were differences in models of care, staffing arrangements, routine practices and physical facilities [3]. Overall, care providers were enthusiastic about postnatal care and committed to ensuring this care was of high quality [4]. However, there was a strong sense amongst the providers interviewed that the provision of hospital based postnatal care is generally considered a lower priority compared with the other episodes of maternity care. Other themes that emerged from the review included the busyness and at times chaotic nature of postnatal wards and the lack of flexibility in meeting individual women's needs. Clinicians suggested that it was difficult to tailor care to each woman given the required checking, education and documentation that had to be undertaken during relatively short hospital stays. Staffing constraints and other factors, including visitors, added to the difficulties in providing woman-centred care [2].

In Australia there are two largely separate options for maternity care – public and private, and in Victoria around two thirds of women have public maternity care [5]. Many aspects of care vary by this distinction, particularly length of postnatal stay. In the public system women stay in hospital on average 2–3 days following a normal vaginal birth and around 4 days following a caesarean section, while women who choose private care stay on average 4 and 5 days respectively [5]. Length of stay also varies markedly by hospital category and location. Metropolitan non-tertiary postnatal units have the highest bed occupancy coupled with the shortest length of stay [2]. Length of the hospital stay has declined dramatically since the 1980s in Australia, but research evidence evaluating early discharge is limited. A number of randomised trials have been reported [6-9] yet all have lacked the statistical power to assess important clinical outcomes. In general it is rarely an option that father's have the opportunity to stay in hospital overnight in the postnatal ward – with the exception of Birth Centre models, although very few women in Victoria have access to this model of care.

The concept of going to hospital to have a baby and staying in hospital postnatally is embedded in Australia's culture, with the hospitalisation of childbirth becoming a social norm during the inter-war years. Fisher et al. suggest that childbirth has been constructed as dangerous and uncontrollable [10]. The authors argue that this construction has occurred in conjunction with the medicalisation of birth, and the increasing destruction of women's feelings of competence in relation to birth and caring for a new baby, especially with a lack of culturally stored information. Against this backdrop there is the potential for fear about the health of a new baby, and medical supervision may be considered the only way to ensure that a new baby is safe [11]. Hence a hospital, with 24 hour medical support, is regarded as the appropriate and safe place to be during and after the birth of a baby [10]. Many mothers of women giving birth today experienced postnatal care in the 1970s and 1980s, when a postnatal hospital stay of up to 10 days was normal. Hence, they may be suggesting to their daughters that a long stay is ideal, and that shortening the length of stay is related to issues of cost saving rather than of optimising outcomes for mothers and babies.

In the public system in Victoria, after women are discharged home following their postnatal hospital stay, most receive some routine domiciliary midwifery care. This is not routinely provided for women choosing private maternity care. Until 1998, Victorian public hospitals had responsibility for the provision of postnatal care during the five days following birth [12], but since then the boundaries for hospital responsibility are defined by clinical judgement rather than time:

*Hospitals are required to ensure adequate postnatal care for women, their babies and their families according to clinical and psycho-social needs. This is defined as providing, as a minimum: at least one postnatal home visit or other contact for all women following discharge from hospital; at least two postnatal home visits or more, if required, for women and their babies with diverse needs, such as women with substance abuse issues, newly arrived migrants, young single mothers, and women with disabilities; at least two or more postnatal home visits for women with complications arising from the birth or immediate postnatal period; and discharge planning, including communication of concerns regarding families to the MCH [Maternal and Child Health] service*[13].

In 2005–2006, 92% of women receiving public maternity care were referred to postnatal domiciliary care [14]. Ninety-five percent of all women receive a home visit from the Maternal and Child Health (MCH) Service (a free service for all Victorian families with children aged 0–6 years offering support, information and advice regarding parenting, child health and development; ), however for many women there may be a service gap of a week or more between receipt of a hospital-run domiciliary visit and the first visit from the local government-run MCH Service [15].

The current situation in a number of Victorian hospitals is critical, with women being discharged earlier in a context of very limited evidence of the optimal length of stay, safety or costs, and little or no evaluation of current moves to shorten postnatal hospital stay. In the longer term we aim to use recent Victorian research to explore an alternative approach to early postnatal care, and to systematically implement and evaluate such an approach in a randomised controlled trial. We hypothesise that an alternative approach may significantly improve: women's experiences of postnatal care, breastfeeding and maternal confidence; and not increase rates of depression or decrease women's overall health status or health outcomes for babies. To inform the further development of this research program, we aimed to gain a more in-depth understanding of women's expectations and/or experiences of postnatal care in the early postnatal period, in hospital and at home; and to elicit women's reactions and views to proposed alternative postnatal care 'packages'. This paper reports on the findings of the first aspect, i.e. the exploration of women's expectations and/or experiences of postnatal care.

## Methods

Focus groups were used for this exploratory study in preference to individual interviews, as the synergistic effects of the group setting may elicit ideas and discussion that may not have arisen in individual interviews [16].

### Participants

Focus groups were planned for the catchment areas of the two hospitals that might be involved in a future pilot and/or trial of an alternative approach to postnatal care. An additional regional location, that was considering restructuring postnatal care services, was included on request. We aimed to capture the views of a range of women including: those who were currently pregnant; those who had a relatively new baby; those who were having (or had just had) their first baby; and those who had given birth to more than one baby. We also aimed to include some new fathers. Participants needed to be fluent in English.

In pregnancy clinics, fliers were distributed by a research team member and interested women completed a form with their contact details. To recruit women with new babies, MCH centre team leaders facilitated our approach to women. In some centres, women who had attended for care at the centre during the year leading up to the study were sent a letter (from the MCH centre) inviting them and their partners to participate in the study. In other centres, women who were currently attending new mothers groups were approached by their MCH nurse and invited to participate during one of their scheduled sessions. It was stressed that participation was entirely voluntary. Written informed consent was obtained prior to the commencement of each focus group. Child care was offered to all participants.

### Data collection and analysis

Focus group guides were developed specifically for the study, with slight variations for pregnant and postpartum women. There were two areas of exploration during the focus groups. The first pertained to overall issues around postnatal care. In this half of the discussion the aim was to explore participants' experiences (for women who had already had their baby), or expectations (for those women who were pregnant), of postnatal care in hospital and in the first few days at home; factors that they particularly liked or were unhappy with about their care; their views or expectations on going home from hospital, with an emphasis on length of stay, professional and social support, continuity of care, rest; and to consider whether there were factors that may have made a difference to their experiences. During the second part of each focus group the facilitator presented the group with some alternative postnatal care packages to elicit participants' reactions and views. Findings from the second aspect of the study are reported elsewhere [17].

Focus groups were facilitated by a member of the research team, with one or two additional research team members acting as non-participant observer(s) to record field notes and complete group logistics (ensuring forms were completed, providing refreshments, etc.). All discussions were audiotaped using a digital recorder. Field notes were taken to describe: the demographics of those attending the group (primarily related to parity and public/private status of maternity booking); themes of the group; and body language of the participants.

All discussions were transcribed verbatim and the transcripts checked against the audiotape for accuracy. A thematic network was constructed using electronic and paper copies of the transcripts from the focus groups, as a way of organizing the thematic analysis, providing emerging basic, organizing, and global themes to describe the data [18]. Transcripts were read and reread to gain an overall perspective then this step by step approach used. Firstly, a coding framework was developed to reduce the text to meaningful and manageable parts, then basic themes that emerged from the text were identified; these formed the 'lowest order' of ideas emerging from the text [18]. These basic themes, which on their own provide very little information about the data as a whole were then summarised into more abstract groups, called 'organizing themes' [18] in order to cluster the basic themes together where they related to similar issues. Finally the organizing themes were summarised as overriding metaphors, or global themes to enable us to make sense of the clusters of lower order themes [18]. Thus the global themes are a summary of the main themes, as well as our interpretation of the data from this analysis. Data analysis proceeded with extensive re-readings of hard copies of the transcripts to ensure the texts were fully explored to guarantee emergence of new basic themes. The initial thematic network was derived by SR and JR. Cross-checking of analysis was undertaken with two other members of the research team (DF, JY). Preliminary themes were presented to the whole team with the transcripts for further discussion and agreement.

To maintain participant confidentiality, names of individuals and institutions were not included in transcripts (pseudonyms used as necessary) and in this paper all participants (male and female) are referred to as either women or participants. The exception to this is where male partners are directly quoted – in these instances it is noted in the quote descriptor that it was a partner who had made the comment. Quotes were chosen to best illustrate the themes emerging from data analysis and the speaker was identified only by (where known) parity, rural/urban focus group location, public/private status and if the woman was pregnant or had already had her baby, e.g. (*primiparous, non-metropolitan, public, postnatal*). Where words have been added to quotes to enable reader understanding they are enclosed in square brackets, i.e. [].

Ethics approval for the study was provided by the Research Ethics Committees of La Trobe University, the Department of Human Services Victoria, Mercy Hospital for Women and Barwon Health.

## Results

### Participants

In total eight focus groups and four individual interviews were held between August and October 2006, with a total of 52 participants. Interviews took place on a few occasions where only one participant attended for a focus group or a participant was unable to attend their nominated session but requested inclusion in the study; in these cases the focus group guide was followed for the interview.

The groups included eight pregnant women, of whom seven were pregnant with their first baby. This was less than the number of pregnant women we aimed to include, but despite a number of different strategies they were a difficult group to recruit. Similarly, only two partners were included in our study, despite strategies such as fliers, letters of invitation, and offering groups at different times and locations. The remaining women who participated were in the postpartum period, and for some this was up to 12 months after the birth of their baby. In one mothers' group the women had been meeting since the birth of their first baby, and most had subsequently had another baby. There were 17 participants who had received public pregnancy care, 11 who had private care and for 24 participants we were unable to determine this from either transcripts or field notes.

### Emerging themes

We found that a range of issues influenced women's views of postnatal care and the type of care that they wanted or expected. Two global themes emerged and are discussed more fully below, but related to: *anxiety and/or fear *around the health and wellbeing of the baby, often couched in terms of safety; and *the transition to motherhood and parenting*, with embedded cultural concepts around this, and a strong sense that the needs of first time mothers were different to the needs of women who had already experienced motherhood. Some of the important basic themes that emerged related to: acquiring skills to care for a new baby; consistent advice from health professionals; continuity of care; lack of staff time; and partner involvement during the postnatal hospital stay. The basic and organisational themes were generally inter-woven, relating back and forward to each other. Systemic factors interacted with the global, basic and organisational themes, with a major one being length of hospital postnatal stay; many issues raised by women were talked about in terms of length of stay and how that did or did not help them achieve some of their expectations. The later discussions around specific alternative postnatal care packages raised no new themes in relation to women's expectations and experiences of postnatal care [17].

### Anxiety and/or fear around caring for a new baby

The first global theme was *anxiety and/or fear*, and comments related to this theme were often couched in terms of safety. It was clear from the focus groups that participants were very concerned about the safety of their new babies, and were very aware that they had responsibility for another life. This contributed to a perceived need for constant professional support.

*...I'd care about the fact that there is someone two minutes away if I need them... if my baby stops breathing... that there is someone on hand and the machinery on hand and... I like that assurance that if anything went wrong... someone was there (primiparous, metropolitan, postnatal)*.

This theme of anxiety and/or fear contributed to women's thinking and responses on a range of issues, particularly length of postnatal stay. That is, a longer length of stay in hospital gave more opportunity to receive 24 hour medical/professional support. Participants viewed domiciliary visits as important, but it was generally agreed that they did not replace the care available in hospital.

*I like that assurance that if anything went wrong... someone was there (primiparous, non-metropolitan, private, postnatal)*.

Having a midwife physically available was also seen as desirable in order to obtain appropriate care and support – and this 24 hour physical access to midwives was preferable compared to domiciliary midwifery care.

*The fact that 24 hours a day there was someone if I needed them I mean even if I had to wait for half an hour just knowing that you know had to wait till three o'clock or whatever their [the domiciliary midwife] appointment was that would just [be] horrific (primiparous, metropolitan, private, postnatal)*.

This physical presence appeared to be reassuring, as way of allaying some anxiety.

*I like that assurance that if anything went wrong... someone was there (primiparous, non-metropolitan, private, postnatal)*.

Another factor related to anxiety and/or fear was a perception for some women that it was not safe to leave hospital too early as they would not know how to care for a new baby, or might not recognise what was normal in terms of their baby's health and behaviour compared to what was not. The days spent in hospital following birth were therefore seen as very important as a means to learning how to care for a new baby, with access to professional support. This was a recurring theme in the focus group discussions.

*I wouldn't get to learn everything I needed to know [in one day] (primiparous, metropolitan, public, antenatal)*.

The thought of leaving hospital earlier than they had experienced or expected contributed to some women feeling quite anxious, even fearful.

*I am six months pregnant and have heard a rumour that hospital stays will be two nights only [soon]. Being a first time mother I find this a little overwhelming. I feel anxious and slightly nervous that I won't feel confident with what I have to do. I believe all the books in the world can't compare to the help, advice and support from midwives and staff in the hospital. Please keep it three nights for first time mums!!! Please! (primiparous, metropolitan, public, antenatal)*.

Participants also expressed concern about being 'forced' out of hospital before they felt confident looking after their baby; for them it seemed like there was no choice but to be in hospital; they felt they needed to be there.

*If I'm in hospital I'm there because medically I need to be there or you know there are issues that are gonna [sic] come up (primiparous, non-metropolitan, postnatal)*.

Despite participants viewing domiciliary visits as important, it was generally agreed that they would not/did not replace the care given in hospital – having a midwife physically available was seen as desirable to provide safety as well as 'care'.

*[Midwife] visits are good, but not really that important. You can always call the hospital or see the GP, [but this] doesn't replace having a midwife on call (primiparous, metropolitan, public, postnatal)*.

Even if a known midwife was to provide the domiciliary care the thought of home care compared to in hospital care appeared anxiety-producing.

*[Having a known midwife at home]...is better than [option of an unknown midwife]... [but] one night in hospital is still a bit scary (primiparous, metropolitan, public, antenatal)*.

### Transition to motherhood and parenting

The concept of *transition to motherhood and parenting *was the other global theme, and one that emerged in each area of discussion. Within this theme there were the cultural concepts about motherhood and parenting, as well as a strong sense that the needs of first time mothers were different to the needs of women who had already experienced motherhood. There were many aspects of the transition to motherhood that emerged, and the concept was particularly evident in discussion of women's expectations of postnatal care and what they perceived they needed in relation to this, i.e. breastfeeding support and education; professional support while acquiring new skills; and the opportunity to rest and be 'cared for' during this transitional time.

### Breastfeeding

Women felt that breastfeeding was something that they struggled with at home and was a skill that ideally needed to be learnt before hospital discharge. Women saw the acquisition of this skill as a factor in their conception of optimal length of postnatal hospital stay.

*I think probably the number one thing for me is that I can breastfeed, that the baby's latching on, I know what to listen for, like I read the books and gotta [sic] have the suck, swallow,...and I'd like to be confident that I knew that definitely (primiparous, metropolitan, public, antenatal)*.

They often used ability to breastfeed, or their milk coming in, as a metaphor for when they would be happy and confident to go home.

*One night is just, just doesn't seem like a long enough time, your milk hasn't come in, you know (primiparous, metropolitan, public, antenatal)*.

### Professional support

As part of gaining confidence in caring for their babies, and learning how to be mothers, women expressed views that it was better for them to do this in a context of constant professional support, or, at a minimum, the constant availability of support. Most felt this was better achieved in the hospital environment, particularly for first time mothers. The option of staying in hospital for a few days after the birth was seen as very important, as it gave women a chance to learn what to do with their new baby while having physical access to professional support.

*One of the reasons we went private was because it was a longer stay and we didn't feel like two nights was adequate preparation to learn to take care of a child ... but I could imagine that with your second child you might want to stay shorter in total...(primiparous, metropolitan, private, postnatal)*.

Women also placed a great importance on being 'cared for' in hospital.

*...it was just really really secure I was a little bit nervous about going home but I could have stayed and lived there forever (primiparous, non-metropolitan, public, postnatal)*.

However despite the general view that constant professional support ensured the safety of their new baby and provided 'care', postnatal participants reported both positive and negative experiences of their hospital postnatal stay. Women's negative views were driven by a gap between expectation and experience of availability of staff time, continuity of care, consistency of care, and communication from hospital staff. In spite of this though, women were positive overall about their own experience, especially relating to partner involvement, visitors, confidence and feeling 'cared for'. Women commented about how good the midwives had been and used words such as "fabulous", "supportive", "fantastic", and "brilliant".

Hospital was paradoxically experienced as noisy and chaotic by some women, e.g. when in a shared room:

*I was uncomfortable, I didn't sleep, the girl next me had a baby that was crying all night (multiparous, non-metropolitan, antenatal)*.

...yet as isolating and lonely by some that stayed in a private room:

*...people have their private rooms and you don't really get to meet other people (primiparous, metropolitan, private, postnatal)*.

Women felt there was a lack of professional support at times while they were in hospital, commenting that staff were too busy or unavailable to provide the care that they expected:

*I got up there and they left me ... to myself. I had no idea about breastfeeding which was hurting... it took them two hours to get to me (primiparous, non-metropolitan, postnatal)*.

Participants described the frustration they felt at having to explain themselves repeatedly to new staff members and/or receiving contradictory advice from different staff members, and how this impacted negatively on their experience of care.

*...the bad thing was probably every shift of midwives you'd get a different midwife... every time so you had to explain yourself again (primiparous, metropolitan, private, postnatal)*.

Women wanted to have their partners involved in the postnatal experience and expressed pleasure and disappointment according to the level of acceptance of their partner's involvement actually experienced in hospital.

*...it was great having the husbands involved (primiparous, metropolitan, private, postnatal)*.

Not all partners felt well received.

*I guess it sort of felt like ... 'go home', they sort of allow for you to stay there but they assumed that you weren't going to be there so always felt like you were always in the way to me. (primiparous partner, metropolitan, private, postnatal)*.

### First time mothers compared with those who had experience

A recurrent theme related to transition to motherhood and parenting as well as to anxiety and/or fear around caring for a new baby was that parents having their first baby needed different care options, compared to parents having subsequent babies. Participants thought that they would be more confident to leave hospital earlier with subsequent babies, but with the first baby needed the professional care and security of being in hospital. For women who had recently had their first baby, the emerging themes related specifically to their expectations and experiences of being at home in the early postnatal period. These included feeling prepared, having family support, their physical health, anxiety and adjustment to parenting, and the hard work associated with a new baby. For those participants who were currently pregnant and expecting their first baby, themes related to concerns about the availability of support, anxiety, a fear of the unknown and not knowing what to expect.

*I think [for a] second child it would be... not easier [to stay a shorter time] but different because you know what to expect whereas the first one... she's crying and you have no idea what's going on... and stuff like that... (primiparous, metropolitan, postnatal)*.

### The reality of being at home and caring for their new baby

The preparation for, or expectations of, actually being at home with a new baby were a concern for women. A number of first time mothers talked about anxiety about going home after the birth. One woman, pregnant with her first child commented:

*Scary as hell, yeah... so it daunts me ... it's a bit scary, it's not something I'm actually looking forward to, I wouldn't say I'm excited about it you know ...just getting home straight away and the thought of what to pack to go out and how to go out and stuff like that so yeah, a bit scared (primiparous, metropolitan, public, antenatal)*.

These fears were born out by the experience of other postnatal women:

*We couldn't leave the house for eight days we were just completely and utterly shockingly overwhelmed for whatever reason I think back now just the thought of taking him out and driving him somewhere we were just... I don't know overwhelmed is the only word I can think of... everything was just anxious (primiparous, metropolitan, private, postnatal)*.

For some women who had already had a previous baby there was also anxiety based on their previous experiences at home after the birth:

*...last time I had a lot of anxiety... ...lot of panic attacks (multiparous, non-metropolitan, antenatal)*.

Being prepared for the reality of parenting was talked about in terms of expectations as well as experiences:

*Yes pretty hard work a lot more than I thought it would be I expected it to be a lot easier especially the feeding (primiparous partner, metropolitan, private, postnatal)*.

*.....how do you prepare for something that you've got no idea what it's gonna (sic) be like (primiparous, non-metropolitan, antenatal)*.

Physical pain, and the problems of managing pain, was also raised as an issue related to being at home versus in hospital, although women differed in their preferred location of care for managing pain:

*I was a little bit apprehensive about going home just because I was still in a bit of pain and couldn't walk around a lot (primiparous, non-metropolitan, postnatal)*.

*I [didn't] want to be in the hospital...by the next morning I'd just had enough and I wanted out... I couldn't get Panadol [paracetamol] when I wanted it, I ... had a lot of pain ..... and I couldn't get my wheat bags heated up because I wasn't allowed to use the microwave, the nurses were too busy, had to wait like half an hour, I had my mother and my partner at home.... So it was just ridiculous, I don't need to be in hospital, I want to go home and I discharged myself at eight o'clock at night and said 'I'm going home' ... I'd rather be in my bed with my pillows and my blankets and my shower, I'm having Panadol when I want it, not having to record how much (primiparous, non-metropolitan, public, postnatal)*.

Participants were concerned about the lack of professional support that they would receive at home, despite many also experiencing a lack of professional support available in hospital. Women saw continued access to midwives, through domiciliary midwife visits and/or other means, as an important aspect of professional support following discharge. However many of the women in the groups who had given birth in the private sector had not experienced domiciliary visits.

*... [I did not experience domiciliary midwife care]... but if that was available, that would be great, you know, just the questions I have that obviously are not going to come up until they get home, I would ...they would be able to answer them for me, even a help line you know, I could phone them up and go 'what do I do?' you know, or something like that, just like a connection or some sort so I don't feel like I've suddenly left hospital and now you're on your own and that's it (primiparous, metropolitan, public, antenatal)*.

Women also raised the importance of adequate levels of social (mainly family) support when they first arrived home from hospital, so that they could concentrate on caring for their baby.

*I think virtually for the first eight weeks I didn't do much else but feed... mum did the grocery shopping or [partner] did the grocery shopping, mum did the washing... so I was fortunate, my parents live up the road... (primiparous, metropolitan, private, postnatal)*.

Other women did not have access to this type of support.

*"No [I won't have support when I go home]... I don't know anyone in Victoria I [only moved here] about five months ago..." (multiparous, metropolitan, public, antenatal)*.

Not all comments about being at home were negative, and for some women it was better to be at home rather than hospital:

*I [didn't] want to be in the hospital... [I] delivered at night so I had that night and I had a day and a night and by the next morning I'd just had enough and I wanted out... (primiparous, non-metropolitan, public, postnatal)*.

## Discussion

We aimed to determine women's views and experiences of postnatal care, and explore their expectations and preferences for care. Women in the focus groups were engaged in the process and were happy to discuss their views and experiences in detail.

A common view expressed by women was that they wanted to stay in hospital after they had given birth until they were confident to look after their baby at home. They wanted to use their time in hospital to learn skills relating to breastfeeding and the general care of their new baby, and felt this would increase their confidence. There was a perception that a required part of learning infant feeding skills was a length of postnatal hospital stay that went beyond the time that breast milk had 'come in'. Participants did not want to leave hospital without learning these skills, although they were generally aware of, and willing to attend, classes/support services following discharge (for example breastfeeding clinics). The global themes *'anxiety and/or fear'*, and '*transition to motherhood and parenting'*, encompass these views. Embedded within these themes was the issue of the needs of first time mothers compared to the needs of women who already had experienced motherhood, as well as the importance of individualised care.

These themes are echoed in the national and international literature. Several papers note the continued emphasis on the attainment of infant care skills and the receipt of information in the relatively short postnatal hospital stay, reporting that this is what women expect to receive and that their carers expect to provide [20,21]. It may be that views such as these are a consequence of the medicalisation of childbirth; that many women believe they need health care professionals around them in order to allay their anxieties or fears during their transition to motherhood and parenting. On the other hand it may be more a reflection of the fact that in most developed countries many women have very little contact with babies prior to becoming a parent; the opportunities to learn from others is limited. Further, many women lack support when they return home with their new baby.

The issue of inflexible length of stay not adapted to individual women's needs has also been raised elsewhere [22]. Ten years ago the UK Audit Commission [23] cautioned that the length of time women spend in hospital should not be defined or standardised on birth outcome, suggesting instead that policies be flexible and that it was essential that women were consulted about the length of stay and the nature of their care.

For those who have never experienced the reality of a hospital stay following birth the idea that a few days in a postnatal unit can instill confidence is obviously a factor in these findings. Interestingly, women who participated in the last Victorian survey of recent mothers and who stayed longer in hospital were somewhat less likely to be confident about caring for their baby when they left hospital [24]. First-time mothers were less likely to be confident about caring for their baby on leaving hospital than mothers of older children. However, first-time mothers who left hospital soon after the birth did not differ from those who stayed longer in relation to confidence about for caring for their baby once they were home [24].

Some participants in the current study reported experiencing care they considered inadequate while they were in hospital. Issues raised included not receiving the expected level or quality of professional care, inconsistency of advice, and a lack of encouragement of the involvement of their partner. These experiences echo those of women who have participated in state-wide surveys of new mothers who reported relatively poor experiences of postnatal care [1] as well as those reported in a Swedish paper [25]. However, negative experiences or perceived deficiencies in care did not result in women wanting to leave hospital earlier. These findings suggest that for many women, being in hospital for the days following the birth is perceived to be the only safe option for their baby. It may be that women experiencing a different system of care such as that in the Netherlands, where there is a system of maternity care assistants who support women for up to eight days might not feel the same way [26]; they may feel more confident to go home sooner after the birth. No such system exists in Australia currently, and to implement a major change such as this, even in an evaluative framework, would require a change in how funding was provided.

The participants in this study were generally of the view that there should be different care options available for first time mothers compared to women who had already had a baby. Women having their second or later child were seen as less in need of postnatal support and education. This view was matched by women's experience of greater confidence looking after subsequent children and less need for the security and constant professional support that they felt hospitals provided. Women who had given birth to only one baby did not want to consider experiencing different postnatal care to what they had received, which may be related to the concept of 'what is must be best' [27], whereas women who had given birth to two or more children were more open to receiving care that was different to what they had previously experienced. This suggests that there is a need for flexibility in postnatal care options, with acknowledgement of each woman's needs, and how these may change with parity and prior experience.

When participants reflected on the experience of the early days at home with a new baby, very few comments were made on domiciliary midwifery care. A more thorough exploration of the organisation and experience of domiciliary care was not within the scope (or focus) of this study. Little is known about the content of domiciliary visits or whether these visits meet the needs of women and their families. Careful planning and evaluation of new approaches to postnatal care need to be taken to ensure that the problems that besiege hospital postnatal care are not simply transferred to the care that women receive at home.

Recruitment of women for the focus groups, in both maternal and child health centres and hospitals, was challenging. It was most successful where a personal relationship either existed or was established between one of the researchers and a maternal and child health nurse who ran a mothers' group. In the groups where a researcher directly approached a maternal and child health nurse, and the nurse took an active interest in the project, the women from their mother's group were more likely to attend the focus group. When the researchers liaised with the maternal and child health manager, and the nurse was then approached by the manager, recruitment was less successful. Recruitment of pregnant women took place in two separate hospitals, through individual face to face methods. It was difficult to recruit an adequate number of women who were currently pregnant to form a focus group. Alternative strategies for recruitment of this group of women need to be explored in future work in this area. The small numbers of pregnant women that were included, as well as the fact that these women were drawn from only two health services limits the generalisation of the results to all antenatal women.

We also experienced difficulty in recruiting women who had experienced public care or who represented the diversity of 'patients' that would be found in the public hospital system in Victoria. Many of the participants had chosen private maternity care, so may be different to women choosing public care. When this became evident, we targeted recruitment at maternal and child health centres in geographic areas that contained more women who had experienced public care. When analysing the results across the whole sample, we found that opinions from focus groups were broadly similar between groups formed primarily of women who had experienced public care and those formed primarily of women who had experienced private care.

We acknowledge both the benefits and limitations of the focus group methodology. Women recruited through the MCH Centres, were predominantly members of established new mothers group. We were mindful of the group dynamics where participants were known to each other. Due to resource constraints we were unable to recruit women who didn't speak English. It may well be that the expectations and experiences of migrant women might differ from the women participating in this study.

When developing postnatal care options for women, it is important to consider women's individual needs and the current norms of society, in terms of the meaning or reasons for hospitalisation during and after childbirth. The results of this research suggest that there needs to be adequate antenatal support and preparation, a focus on individuality, and some flexibility built into postnatal care options. Previous research has reported that postnatal care providers also believe that postnatal care should be flexible and individualised, although there are many structural barriers that may prevent this from occurring [3].

## Conclusion

The women in this study were generally concerned about the safety and wellbeing of their new baby. They expressed a lack of confidence in themselves as new mothers and their ability to care for their baby without professional support. There was a consistent view that the physical presence and availability of professional support helped alleviate these concerns, and this was especially the case for women having a first baby.

It seems likely that there are a number of societal factors contributing to women's views of hospital as the safest and most appropriate place to be after giving birth. These include: the social construction of childbirth in Australia within a medical model; the medicalisation of pregnancy and birth; and the fact that many women giving birth at this point in time have limited exposure to babies or birth. This in turn relates to our changing social demographics – the reduced size of families, advanced maternal age, and women's increased participation in the paid workforce. There is little opportunity for birth and mothering to be learned and internalised as 'normal' (or natural).

Given this situation, women understandably have concerns about any moves to shorten postnatal lengths of hospital stay. It is crucial that women's concerns and needs be considered when service delivery changes are planned. Hence it is important that any move towards a shorter postnatal length of stay is evaluated, in terms of the physical and mental health of both mother and baby, and the mother's satisfaction with the care received. If alternative arrangements for the provision of postnatal care are introduced, evaluation should be undertaken and include an examination of the impact on the health of women and babies, including health outcomes as well as parental anxiety and confidence; the views of women and service providers; and the economic impact on health services and women and their families.

## Competing interests

The authors declare that they have no competing interests.

## Authors' contributions

Study conception: DAF, HMcL, JY. Study design: DAF, HMcL, JY, JR, LG. Initial grant application: DAF, HMcL, JY, JR, LG. Questionnaire development, piloting and completion: DAF, HMcL, JY, JR, LG, SR. Coordination and implementation of study: SR, DAF, HMcL, JR. Data collection, analysis and management: SR, DAF, JR, JY, HMcL. Drafted manuscript: DAF. All authors read and approved final manuscript.

## Pre-publication history

The pre-publication history for this paper can be accessed here:


